# Ultrasonographic prevalence and characteristics of non-palpable thyroid incidentalomas in a hospital-based population in a sub-Saharan country

**DOI:** 10.1186/s12880-017-0194-8

**Published:** 2017-03-04

**Authors:** Boniface Moifo, Jean Roger Moulion Tapouh, Sylviane Dongmo Fomekong, François Djomou, Emmanuella Manka’a Wankie

**Affiliations:** 1 Department of Radiology and Radiation Oncology, Faculty of Medicine and Biomedical Sciences, The University of Yaounde 1, Yaounde, Cameroon; 2 Radiology Department YGOPH, Yaounde Gynaeco-Obstetric and Pediatric Hospital, PO Box 4362, Yaounde, Cameroon; 3 Yaounde University Teaching Hospital, Yaounde, Cameroon; 4 Douala General Hospital, Douala, Cameroon

**Keywords:** Thyroid incidentaloma, Thyroid nodule, TIRADS, Prevalence, Sub-Saharan country

## Abstract

**Background:**

Thyroid incidentalomas (TI) are highly prevalent asymptomatic thyroid nodules with ultrasound as the best imaging modality for their detection and characterization. Although they are mostly benign, potential for malignancy is up to 10–15%.

In sub-Saharan Africa little data exists on the prevalence and risk categorization of TI. The aim of this study was to determine the prevalence and ultrasound characteristics of non-palpable thyroid incidentalomas among adults in sub-Saharan setting.

**Methods:**

A cross sectional study was carried out between March and August 2015, at two university teaching hospitals. Sampling was consecutive and included all adults aged ≥ 16 years, presenting for any ultrasound other than for the thyroid, with no history or clinical signs of thyroid disease, and no palpable thyroid lesion. Ultrasound was done using 4 to 11 MHz linear probes. Subjects with diffuse thyroid abnormalities were excluded. Variables studied were age, gender, thyroid volume, ultrasound characteristics of thyroid nodules, TIRADS scores. Differences were considered statistically significant for *p*-value < 0.05.

**Results:**

The prevalence of TI was 28.3% (126 persons with TI /446 examined). This prevalence was 46.2% in population ≥ 61-year-old; 6.3% in population ≤ 20-year-old; 33.3% for females and 18.4% for males (*p* < 0.001). Of the 241 TI found, 49.4% were cysts, 33.6% solid, 17.0% mixed; 37.8% <5 mm and 22% >10 mm. Solid TI were mainly hyperechoic (42.0%), 3/81 were markedly hypoechoic. Sixty-nine out of 126 persons with TI (54.8%) had at least two nodules. Solitary nodules were predominant in the age group ≤20 years. Of 241 TI, 129 (53.5%) were classified TIRADS 2, 81 (33.6%) TIRADS 3, 25 (10.4%) TIRADS 4A, 6 (2.5%) TIRADS 4B, and none TIRADS 5. Characteristics associated with increased risk of malignancy where mostly founded on solid nodules (*p* < 0.000) and nodules larger than 15 mm (*p* < 0.001).

**Conclusion:**

Thyroid incidentalomas were very frequent with a prevalence of 28.3% and potential risk of malignancy in 12.9%. Prevalence had a tendency to increase with age and in female. Cystic nodules were the most prevalent. Potential for malignancy would be increased for larger and solid nodules.

## Background

Thyroid incidentalomas (TIs) are asymptomatic nodules discovered accidentally during imaging studies indicated for other reasons [1]. Ultrasound is the best imaging modality for the detection and characterization of these nodules [2, 3]. Various studies have reported a prevalence between 50 and 67% [2, 4–7]. They are mostly benign. However, there is a potential for malignancy in less than 10–15% [1, 2, 8], depending on the method of sampling and the characteristics of nodules.

TIs therefore represent a clinical challenge and a source of anxiety to patients. The clinician needs to correctly access the risk of each nodule, in order to correctly determine if and what further investigation is necessary. The TIRADS (Thyroid Imaging Reporting and Data System) permits this classification with recommendations on the need for cytologic verification or ultrasound surveillance [6, 7, 9, 10].

In sub-Saharan Africa little data exists on the prevalence and risk categorization of TI [11]. The aim of this study was therefore, to determine their prevalence in the adult population, and describe their ultrasound characteristics based on TIRADS.

## Methods

A cross-sectional study was carried out in two university teaching hospitals from March to August 2015.

### Study population

Persons aged ≥ 16 years, referred to the diagnostic imaging department for ultrasound scans other than that of the thyroid, who accepted freely to participate in the study were included. They had no history, palpable or other clinical signs of thyroid disease. A consecutive non-probabilistic sample was taken. Patients with diffuse thyroid disease were excluded. Verbal informed consent from participants was required.

### Thyroid ultrasound procedure and image interpretation

The ultrasound was done free of charge using the routine procedure [12], with linear probes of 4–11 MHz frequency. The machines used were *Prosound alpha 6* (Hitachi Medical Europe, France) 2015 and *SSI-8000* (Sonoscape Co Ltd, China) *2013*. Images were stored in the hard drive of each machine. An initial interpretation was done by the operator during the course of the scan. A second reading was done later by the operator and two radiologists with at least five years’ experience in thyroid sonography. Nodules were classified by consensus according to TIRADS (Table 1), as proposed by Russ and al [9, 13].

**Table 1:** TIRADS classification according to Russ and al [9, 13]

TIRADS	Signification	Ultrasonographic characteristics	Malignancy risk (%)
TIRADS 1	Normal thyroid	▪ Normal thyroid US	-
TIRADS 2	Benign aspects	▪ Simple cyst ▪ Spongiform nodule ▪ ‘White knight’ aspect ▪ Isolated macrocalcification ▪ Typical sub-acute thyroiditis	0.0
TIRADS 3	Probably benign aspects	▪ None of the high suspicious aspect ▪ Isoechogenic ▪ Hyperechogenic	0.25
TIRADS 4A	Low suspicious aspect	▪ None of the high suspicious aspect ▪ Moderately hypoechogenic	6.0
TIRADS 4B	High suspicious aspects with 1 or 2 signs and no adenopathy	▪ Taller-than-wide shape ▪ Irregular or microlobulated margins ▪ Microcalcifications ▪ Marked hypoechogenicity	69.0
TIRADS 5	High suspicious aspects with ≥ 3 signs and/or adenopathy	▪ Taller-than-wide shape ▪ Irregular or microlobulated margins ▪ Microcalcifications ▪ Marked hypoechogenicity	100

A preconceived data collection form was filled out for each subject. Variables studied included: age, gender, thyroid volume, ultrasound characteristics of any nodules found (echogenicity, calcifications, borders, height/breadth in transverse plane), and TIRADS score for each TI. The CSPro 5.1 software was used to create the data entry mask anonymously. Stata version 11 and SPSS version 18 software enabled the analysis of the data. The chi square test assessed the association between the TIRADS score and various socio-demographic and sonographic characteristics. The comparison of the prevalences were performed using the Fisher exact test. Results were expressed in numbers and percentages for categorical variables. Differences were considered statistically significant for *p* < 0.05.

## Results

Four hundred and forty six subjects aged 16 to 89 were included, with 294 females (65.9%; sex ratio F/H = 1.9). The most frequent age groups was 21–40 years with 48.7% (Table 2).

**Table 2:** Prevalence of thyroid incidentalomas (TIs) with respect to age and gender

TIs	Female		Male		Total	
Age (years)	Population	n (%)	Population	n (%)	Population	n (%)
≤20	13	1 (7.7)	3	0 (0.0)	16	1 (6.3)
21–40	163	35 (21.5)	54	2 (3.7)	217	37 (17.1)
41–60	90	43 (47.8)	71	21 (29.6)	161	64 (39.8)
≥ 61	28	19 (67.9)	24	5 (20.8)	52	24 (46.2)
Total	294	98 (33.3)	152	28 (18.4)	446	126 (28.3)

(*p* < 0.001)

Of the 446 individuals included, 126 had thyroid incidentalomas giving a prevalence of TIs of 28.3%. This prevalence was 33.3% among women (*p* < 0.001) and 46.6% in individuals aged more than 60 years (Table 2).

### Characteristics of incidentalomas

**Fig. 1:**
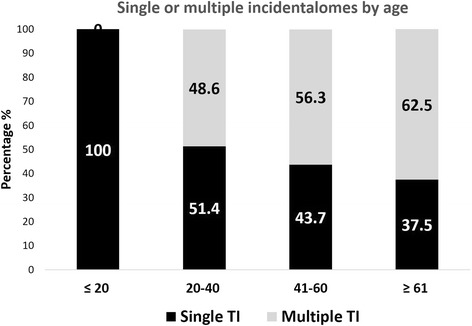
Distribution of multiple incidentalomas according to age

The 126 subjects with TI cumulated a total of 241 TIs with 69 individuals (54.8%) having two or more TI (Fig. 1). Multiple TI was found in 57.2% of females and 42.2% of males.

The location of TI was 54.0% (130/241) in the right lobe and 3.7% in the isthmus (9 cases). Most were inferiorly (base) located 38.2% (92/241), followed by location within the corpus (31.1%).

Of the 241 TIs, cystic nodules accounted for 49.4%; solid nodules for 33.6%; mixed (cystic and solid) for 17.0%. According to size 37.8% TIs were <5 mm and 22% >10 mm (Table 3).

**Table 3:** Distribution of incidentalomas with respect to size and tissue structure

Thyroid incidentalomas	Cystic	Solid	Mixed	Total
Largest diameter	n (%)	n (%)	n (%)	n (%)
< 5 mm	67 (73.6)	11 (12.1)	13 (14.3)	91 (37.8)
5–9	41 (42.3)	36 (37.1)	20 (20.6)	97 (40.3)
10–14	8 (22.9)	23 (65.7)	4 (11.4)	35 (14.5)
≥ 15	3 (16.7)	11 (61.1)	4 (22.2)	18 (7.5)
Total	119 (49.4)	81 (33.6)	41 (17.0)	241 (100)

In regard to solid nodules (81/241), 42.0% were hyperechoic, 29.6% isoechoic, 24.7% with moderate hypoechogenicity and 3.7% were markedly hypoechoic. 39 solid TIs had a peripheral hypoechoic ring.

Solid and mixed incidentalomas had well defined borders in 91.0% of cases; ten nodules (8.2%) had indistinct borders. Eight nodules had macrocalcifications, two had microcalcifications. Half of them did not show any form of vascularization on Doppler examination (pulse and power). Peripheral vascularization was seen in 39.3% of cases (48/122). Only one nodule presented predominantly central vascularization.

### TIRADS classification of TIs

**Fig. 2:**
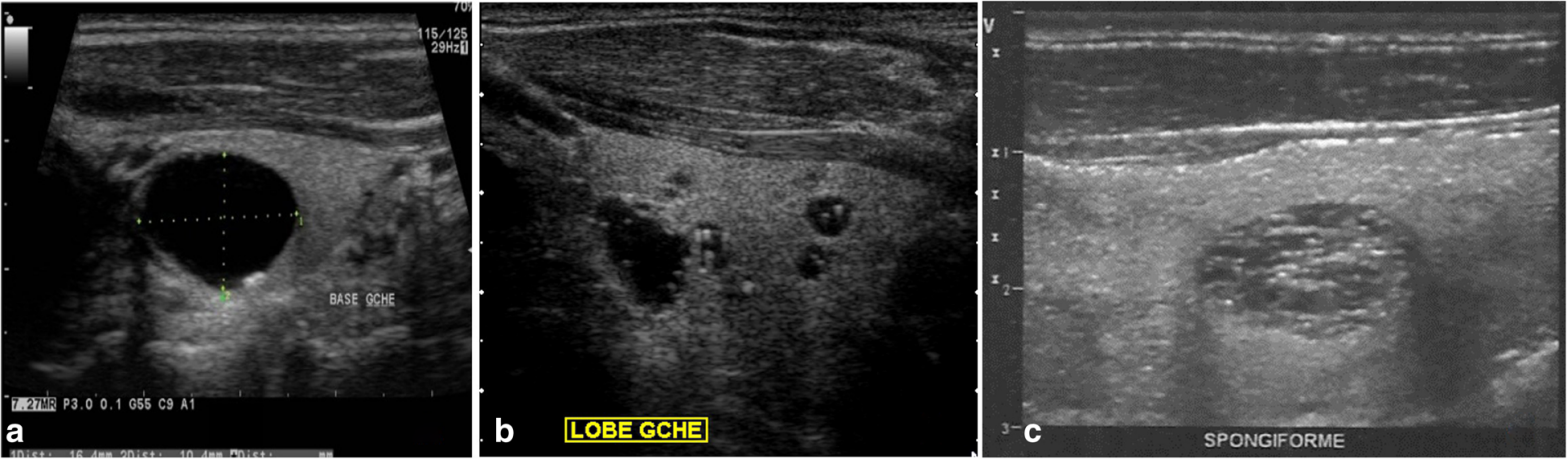
TIRADS 2 thyroid incidentalomas. Anechoic cyst with small parietal calcification (**a**). Colloid cysts of the left lobe (**b**) cystic formations containing hyperechoic pits with comet tail artefacts characteristic of colloid granulations (**b**). Spongiform nodule of the right lobe (**c**)

**Fig. 3:**
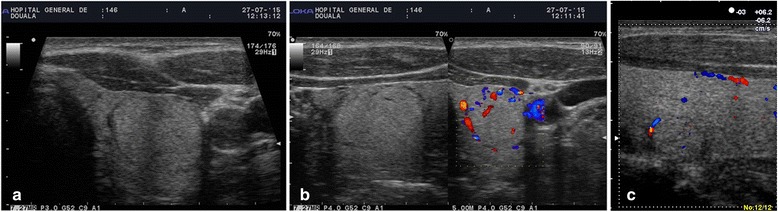
TIRADS 3 thyroid incidentalomas. Hyperechoic nodule with sharp margins, peripheral hypoechoic halo and peripheral vascularization on the left lobe (**a**, **b**). Isoechogenic nodule with peripheral hypoechoic halo and peripheral vascularization (**c**)

**Fig. 4:**
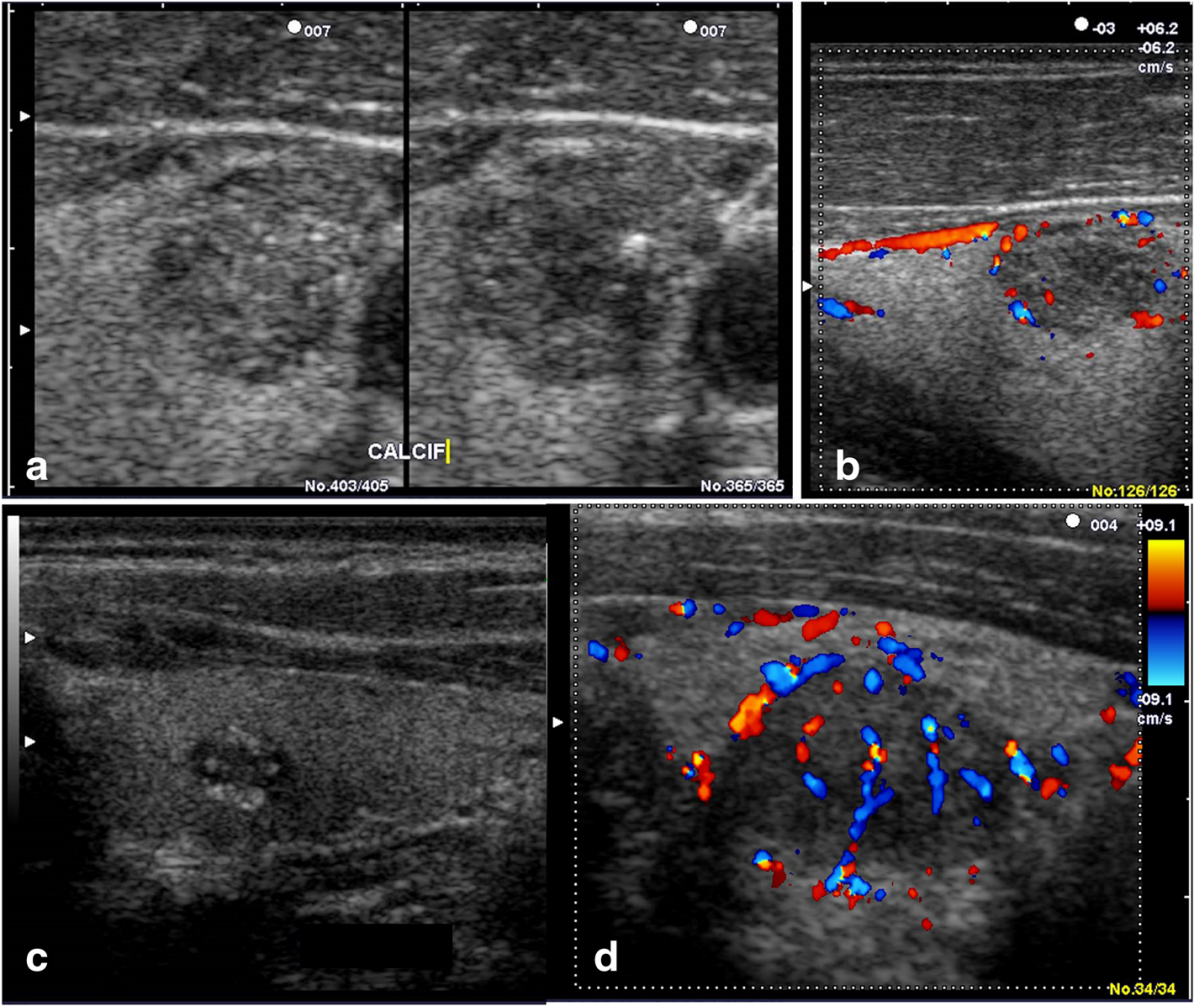
TIRADS 4 incidentalomas. TIRADS 4A: a moderately hypoechoic solid nodule of sharp margins (**a**) with peripheral vascularization (**b**). TIRADS 4B: hypoechoic nodule with microcalcifications (**c**) and marked hypoechoic nodule with mixed vascularization (**d**)

Of 241 TIs, 129 (53.5%) were classified TIRADS 2 (Fig. 2), 81 (33.6%) TIRADS 3 (Fig. 3), 25 (10.4%) TIRADS 4A, 6 (2.5%) TIRADS 4B (Fig. 4), and none TIRADS 5.

Of the 31 TIRADS 4 thyroid incidentalomas, 77.4% were solid and 22.6% mixed (*p* < 0.000). None was cystic (Table 4); 14.3% of TIRADS 4 nodules were 10–15 mm, 22% were > 15 mm.

**Table 4:** TIRADS classification of thyroid incidentalomas with respect to tissue structure

Thyroid incidentalomas	Cystic	Solid	Mixed	Total
Classification	n (%)	n (%)	n (%)	n (%)
TIRADS 2	116 (48.1)	1 (0.4)	12 (5.0)	129 (53.5)
TIRADS 3	3 (1.3)	56 (23.2)	22 (9.1)	81 (33.6)
TIRADS 4A	0 (0.0)	20 (8.3)	5 (2.1)	25 (10.4)
TIRADS 4B	0 (0.0)	4 (1.7)	2 (0.8)	6 (2.5)
Total	119 (49.4)	81 (33.6)	41 (17.0)	241 (100.0)

(*p* < 0.001)

There was no significantly strong association between age, gender and TIRADS score. Prevalence of potentially malignant nodules (TIRADS 4) increased with size (*p* < 0.001): Table 5.

**Table 5:** Prevalence of TIRADS 4 thyroid incidentalomas with respect to size

Size	< 5 mm	5–9 mm	10–15 mm	>15 mm	Total
n (TIRADS 2, 3 and 4)	91	97	35	18	241
TIRADS 4A	8	11	3	3	25
TIRADS 4B	1	2	2	1	6
Malignant potential (4A + 4B)	9.9%	13.4%	14.3%	22.2%	12.9%

(*P* < 0.001)

## Discussion

The aim of this study was to determine the prevalence and ultrasound characteristics of thyroid incidentalomas, amongst adult in a hospital based setting. The prevalence of TIs was 28.3% with 49.4% being cysts, and 87.1% classified TIRADS 2 or 3.

### Population

Our population is comparable to those studied by Papini and al in Italy [2] and by Kim and al in Seoul [14], with female predominance. In our study women represented more than 2/3 of the population. This could be explained by the higher proportion of women referred to the imaging department, as well as in the general population in our setting.

The mean age in our study was 42 years. Papini and Kim [2, 14] had higher mean ages of 47.8 and 49.2 respectively.

### Prevalence of thyroid incidentalomas on ultrasound examination

In our series, the prevalence of TIs was estimated at 28.3% compared to 27% in southern Finland [15]. This is slightly higher than the 22.4% reported by Olusola-Bello in Nigeria [11] and 21% by Kamran and al in Karachi, Pakistan [16], both in 2014. In 2009, in Germany, Guth and al [17] reported 68% prevalence with 53% < 5 mm. The prevalence therefore varies with age, sex, technology available (operator, probe frequency), the minimum size of nodules, and the presence or absence of iodine deficiency in the population. High resolution machines now permit the detection of much smaller nodules, a few millimeters in size [12].

Prevalence of TIs was significantly higher in females (33.3%; *p* < 0.001), than males (18.4%). This has been also reported by authors in Nigeria [11], Pakistan [16] and Iran [18]. It is generally known that the prevalence of TIs amongst females is four times that of males [10]. This prevalence increases with age, with maximum prevalence in persons > 60 years. TIs are considered as part of the physiologic aging process of the thyroid gland [10, 11, 15, 16]. This might also explain the increase in prevalence with age in our population.

### Ultrasound characteristics of thyroid incidentalomas

The locations of TIs had a tendency to be in the right lobe (54%). This result was similar to the previous reports [2, 11, 16]. It might be explained by the difference of the native sizes between right and left lobes, that the right lobe was supposed to be 1.2 folds larger than the left [19–21].

Almost half of TIs were cystic in nature (49.4%) in line with other studies [7, 10, 11]. Solid nodules (33.61%) were mainly hyperechoic (42.0%). Other authors found a predominance of isoechoic or hyperechoic nodules too [2, 7, 14, 18, 22]. Characteristics to indicate malignant potentials were rare in our series (one case with lobulated margins, two cases with microcalcifications). Liebeskind and al [23] had similar findings. Different studies showed marked variability in the size of nodules. We found 22% of nodules ≥ 10 mm in our series, compared to 43% by Kamran and al [16] and 66.5% by Kim and al [14]. Fine needle aspiration is usually recommended for nodules ≥ 10 mm; those < 5 mm are usually transitory, difficult to characterize and to aspirate.

### TIRADS classification (Table 1) of thyroid incidentalomas

We had similar prevalence of TIRADS 2 nodules (54.5%) as Olusola-Bello and al (54.0%) in Nigeria [11]; these two populations share similar characteristics. The risk of malignancy in our series was 12.9%, which falls within the range of 7–15% described in existing literature [2, 3, 9, 14]. There was a significant association between risk of malignancy and solidity of nodules (*p* < 0.000): of the 31 nodules classified TIRADS 4, 77.4% were solid and 22.6% of mixed echogenicity. Solidity of nodules is considered suspicious of malignancy in the TIRADS classification, as opposed to purely cystic and spongiform nodules. There is also an association between size and risk of malignancy (*p* < 0.001), the prevalence of potentially malignant nodules increases with size (14.3% of TIRADS 4 nodules were 10–15 mm, 22% were > 15 mm). This reiterates the need for cytological examination of nodules > 10 mm whereas those < 5 mm do not need this [10, 14].

We did not find an association between the TIRADS score and age or sex. Capelli and al did not find a significant difference in the prevalence of malignant nodules among the two sexes [22]. Kim and al [14] reported a significantly higher prevalence among females who however represented 4/5 of their study population.

There were some limitations in this study: first this study was conducted in a hospital-based setting; and second no cytological analysis of nodules was performed because participants were either reticent to do so, or lost to follow up. A community based study may better determine the true prevalence of thyroid incidentalomas in the general population.

## Conclusion

Thyroid incidentalomas were very frequent with a prevalence of 28.3% and a potential risk of malignancy in 12.9%. Prevalence had a tendency to be increased with age and female sex. Purely cystic nodules are the most frequent. Risks of malignancy would be increased for a larger size and would be higher for TIs of solid components.

## Abbreviations

TI: Thyroid incidentalomas
TIRADS: Thyroid imaging reporting and data system
